# Translation and cultural adaptation of EORTC QLQ-LC 29 into Nepalese language for lung cancer patients in Nepal

**DOI:** 10.1186/s41687-020-00205-w

**Published:** 2020-06-17

**Authors:** Sunil Shrestha, Sudip Shrestha, Bhuvan KC, Binaya Sapkota, Anil Khadka, Saval Khanal, Michael Koller

**Affiliations:** 1 Department of Pharmacy, Nepal Cancer Hospital and Research Center, Lalitpur, Harisiddhi Nepal; 2 Department of Medical Oncology, Nepal Cancer Hospital and Research Center, Lalitpur, Harisiddhi Nepal; 3 School of Pharmacy, Monash University Malaysia, Jalan Lagoon Selatan Bandar Sunway, Selangor, 47500 Malaysia; 4 Department of Pharmaceutical Sciences, Nobel College, Affiliated to Pokhara University, Sinamangal, Kathmandu, Nepal; 5 Department of Public Health, Ohm College of Health Science, Affiliated to Purbanchal University, Kathmandu, Chabahil Nepal; 6 Department of Pharmaceutical and Health Service Research, Nepal Health Research and Innovation Foundation, Lalitpur, Nepal; 7 Behavioural Science Group, Warwick Business School, Coventry, UK; 8 Center for Clinical Studies, University Hospital Regensburg, Regensburg, 93042 Germany

**Keywords:** Lung cancer, Nepalese version, Cultural adaptation, LC29, Translation, Validation

## Abstract

**Background:**

The quality of life (QoL) of patients with lung cancer (LC) may be affected by disease-related limitations such as patients’ functioning, the severity of symptoms, financial problems resulting along with the side effects of the treatment. The objective of this study was to translate LC-specific QoL questionnaire EORTC QLQ-LC29 into Nepalese language for Nepalese LC patients.

**Methods:**

In the process of translation, the European Organization for Research and Treatment of Cancer (EORTC) translations guidelines were followed. The translated questionnaire was pilot-tested in a sample of 15 patients with LC. Descriptive statistics were calculated with SPSS version 21.0.

**Results:**

All steps of the EORTC translation guideline were followed successfully. Fifteen lung cancer patients were included in the pilot study. Sixty percent were male and the mean age was 49.87 (range 21–76 years**).** For all items not related to thoracic surgery, patients used the entire range of the response options from 1 to 4 and no missing responses were observed. The highest mean (indicating a high symptom burden) was observed for the item number. 35 (shortness of breath; Mean = 3.33, SD = 1.11) and the lowest mean for an item number. 45 (dizzy; Mean = 1.73, SD = 0.96).

**Conclusion:**

The Nepalese version of EORTC QLQ-LC29 is a result of a successfully conducted rigorous translation procedure, and is highly comprehensible as well as acceptable to Nepalese LC patients. Thus, the Nepalese version of EORTC QLQ-LC29 is ready to be used in international clinical studies as well as in Nepalese clinical practice.

## Introduction

Lung cancer (LC) is one of the commonest cancers causing a large death all over the world. The incidence of LC was estimated to be 2.1 million, causing 1.8 million deaths per annum [1, 2]. Furthermore, almost two-thirds of the global LC incidence and mortality occur in lower and middle-income countries [3]. In Nepal, cancer is considered as one of the major non-communicable diseases (NCDs) which causes significant social and financial burden [4, 5]. The Global Burden of Disease (GBD) Study 2017 classified tracheal, bronchus and LC as a single group [6], the report states that the healthcare burden and costs associated with this group of cancer were significant on a global scale. Its five-year survival rate is around 18%, which was found to be considerably less than that of other foremost cancers such as breast cancers, cervical cancers, etc. [7].

The GBD study (2017) also estimated that each year 2327 people die due to tracheal, bronchus, and lung cancer with a mortality rate of 7.79/100,000 (global mortality = 24.65/100,000). The estimated prevalence and incidence rates for tracheal, bronchus and lung cancer in Nepal were 7.96 (global prevalence = 43.76/100,000) and 7.41/100,000 population (global incidence = 28.31/100,000) respectively. The total burden associated with this group of cancer in Nepal was 54,760.72 DALYs, which was 0.61% of the total burden of diseases estimated for Nepal [6]. The histological type of LC is poorly studied in Nepal. Currently, there are twelve cancer hospitals provide diagnostic and treatment services for different cancer patients including LC in Nepal. Out of these hospitals, some are government hospitals and others are private hospitals. Radiation therapy, chemotherapy, surgery, immunotherapy, targeted therapy are available treatment options for LC patients in Nepal. However, all these services are not available in all hospitals; only few hospitals (both private and government) out of the 12 cancer hospitals provide comprehensive cancer treatment services for different types of cancer. Patients also use traditional medicines such as herbal and ayurvedic medicines for the treatment of LC in Nepal. However, the exact data on the use of traditional medicines in the treatment of LC is not available.

In an oncology practice and research, the quality of life (QoL) is increasingly being recognized as an essential endpoint and has become a major determinant in deciding treatment options [8, 9]. Out of many research tools for assessing QoL in LC patients, the most often used tool are QLQ-C30 and QLQ-LC13 module which was developed by the European Organization for the Research and Treatment of Cancer (EORTC). EORTC QLQ-C30 questionnaire is a generic questionnaire that assesses general QoL of all cases of malignancy [10] and the LC-specific (EORTC QLQ-LC13) module assesses specific symptoms related to LC and its treatments [11].

Since the QLQ-LC13 was developed and published in 1994 [11], a project which was initiated by the EORTC to update the module to keep pace with the many changes in diagnostics and treatment of LC. The updated version is presently known as EORTC QLQ-LC29 [12], which contains a total of 29 questions that cover new side effects related to targeted therapy and immunotherapy as well as a surgical subscale to be used in patients who underwent thoracic surgery.

Native Nepalese speakers are estimated to be 11,826,953 (i.e., 44.6% of the total population) in Nepal [13]. Around 50,000 of Nepalese language speakers are living in India. Apart from Nepal and India, it is also spoken by a substantial number of people living in Myanmar and Bhutan. In total, there are 15 million native Nepalese language speakers, the Nepalese language use ‘Devanagari’ script when used in written form [14].

Currently, a Nepalese translation is available for the QLQ-C30, but not for the QLQ-LC29. A Nepalese version of LC29 would be helpful to measure QoL in patients who speak Nepalese language all around the world. Therefore, the objective of the current study was to translate the EORTC QLQ-LC29 into the Nepalese language and to pilot-test the translated questionnaire in a small sample of Nepalese LC patients.

## Methods

### Translation procedure

The questionnaire to be translated was the Phase 3 version of QLQ-LC29 which has 29 items aggregated into five multi-item scales (coughing, shortness of breath, side-effects, existential issues related to tumor progression, and surgery-related symptoms). It also has five single items which include coughing up blood, pain in the chest, arm/shoulder and other parts of the body, and weight loss. All items refer to a specific time (i.e., “during the past week”), and are to be scored on a 4-point Likert scale with the response options labeled “not at all”; “a little”; “quite a bit”, and “very much”.

**Fig. 1:**
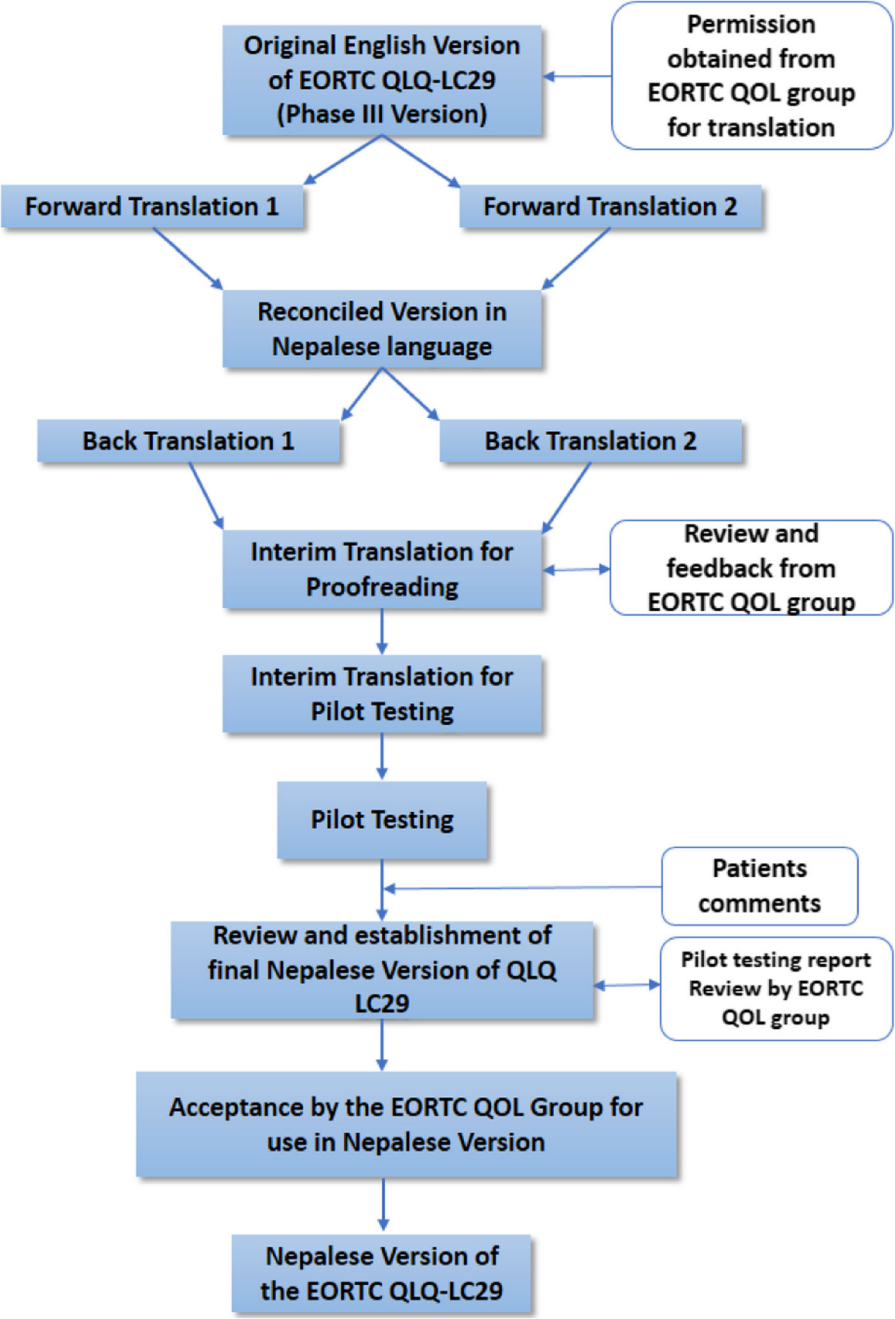
Translation procedure of EORTC QLQ-LC29 into Nepalese version

For the translation of the QLQ-LC29, approval was obtained from the EORTC QoL Department. The translation followed the standard EORTC procedure, including forward translation, reconciliation, back translation, proofreading and pilot testing in a small sample of the target population, whereby 15 patients are considered sufficient [15]. Figure 1 shows the flowchart of the translation procedure of EORTC QLQ-LC29 into the Nepalese version.

### Pilot testing of the Nepalese version of the QLQ-LC-29

#### Procedure

After the Nepalese translation was approved by the EORTC Translationa Unit (TU), it was pilot-tested in a 15 Nepalese patients diagnosed with lung cancer (LC) at an oncology-based hospital situated in Province No 3 in Nepal. The treatment available in this hospital are chemotherapy, radiotherapy, surgery, targeted therapy, and immunotherapy. More than 3000 patients visit this hospital for the treatment of cancer annually.

Before the data collection for the pilot study, formal permission was obtained from each participant and the respondents were informed about the purpose and objectives of the study. Privacy and confidentiality were maintained by not disclosing the name of the participants and ensuring them, that collected information was used only for the research purpose. Patients were handed with the Nepalese version of the QLQ-LC29 along with a self-administered questionnaire (i.e demographic questionnaire such as age, gender, ethnicity, educational qualifications and occupation) by a researcher. Patients filled out both questionnaires themselves. After completion, they were interviewed by the researcher if they had any comments regarding the questionnaire to determine whether translated questionnaire items were either difficult to answer or confusing or difficult to understand or upsetting/offensive. Patients were also asked whether they would have worded the question differently.

#### Patient inclusion and exclusion criteria

Inclusion criteria for this study were histologically confirmed cases of LC diagnosed patients, active cancer treatment, mentally fit to complete a questionnaire, able to understand the Nepalese language and informed consent. We have excluded those patients who were not mentally fit to complete a questionnaire and unable to understand the Nepalese language. There were no restrictions regarding gender, age, or level of education.

#### Statistical analysis

Descriptive statistics included counts, percentages, means and standard deviations and were reported for age, gender, ethnicity, education, and occupation. Qualitative data stemming from patient interviews were listed in tabular form. The IBM Statistical Package for the Social Sciences (SPSS) Version 21.0 was used (IBM SPSS Statistics for Windows, IBM Corp Armonk, NY, USA, 2017).

#### Ethics approval

The ethics approval was obtained from the Institutional Review Committee (IRC) of Nobel College, Affiliated to Pokhara University, Kathmandu, Province No. 3, Nepal (Reference number EPY IRC 216/2018). Permission to conduct the study at the oncology-based hospital was also obtained from the respective Department of Medical Oncology.

## Results

### Translation procedure

#### Forward translations

The EORTC QLQ LC-29 was initially translated from English into Nepalese and a version that would be conceptually and culturally as close as possible to the original English version of QLQ-LC-29. Two forward-translators (FT1 and FT2), native speakers of the Nepalese languages and fluent in English, provided their translations independently. FT1 is Health Economist and Behavioral Scientist with Nepalese as the native language and had very good command over English whereas FT2 is a Lecturer of Clinical Pharmacy working at one of the colleges of Nepal who also is a native Nepalese speaker and had very good command over English. Both the translators were knowledgeable about clinical terminologies, phrases, slang, and jargon used in Nepalese and English language.

#### Reconciled version

The two forward translations by FT1 and FT2 were merged into one reconciled version by the Translation Coordinator (TC) whose objective was to choose or build from the two forward translations an optimal translation of each item.

#### Back translations

The reconciled Nepalese questionnaire was handed over to two back translators (BT1 and BT2), who were requested to produce an English translation of the Nepalese questionnaires in simple, comprehensible language. BT1 is a senior consultant medical oncologist working at a cancer hospital in Nepal who has strong command on both English and Nepalese language, whereas BT2 is a Nepalese clinical pharmacist and academician working in a pharmacy school in Malaysia with strong command in both Nepalese and English languages. Both back-translation by BT1 and BT2 were compared with the original English version by TC and consultant medical oncologist and reconciled into one version. A back-translation report was then sent to EORTC for review and approval. The EORTC Translation Coordinator sent some comments and sought some clarifications, according to which the translations were slightly modified.

#### Proofreading

The preliminary translation is then sent to a professional proofreader by TU. After proofreading, the TU sends the report to the TC for approval. After agreement in wording, TU prepared the interim translation for pilot testing.

### Pilot study

#### Patient characteristics

The interim translated Nepalese questionnaires for the pilot study were administered to the fifteen patients who were diagnosed with LC. The study population consisted of participants who were native Nepalese speakers. Medical records were retrieved for the clinical status of patients and only patients who were diagnosed as LC was included for a pilot study.

Fifteen patients were enrolled in the present study; the mean age of the entire cohort was 49.87 (standard deviation 16.89, range 21–76 years) (Table 1). Sixty percent of the participant (*n* = 9) were male. Fifty-three percent of the respondents have attended university/college. All recruited patients accepted and answered self-administered questionnaires (i.e. demographic questionnaire) and Nepalese LC29 developed for pilot study.

**Table 1:** Patient Characteristics (*n* = 15)

	Respondents’ characteristics (***n*** = 15)	Frequency (%)
Age	Mean ± SD: 49.87 ± 16.89 years; range: 21–76 years	
Gender	Male	9 (60%)
	Female	6 (40%)
Ethnicity	Brahmin / Chhetri	5 (33.3%)
	Newar	6 (40%)
	Tamang	1 (6.7%)
	Madhesi	2 (13.3%)
	Others	1 (6.7%)
Educational Level	No formal education / No Schooling	3 (20.0%)
	Primary and Secondary School	4 (26.6%)
	College / university	8 (53.3%)
Occupation	Not working/ Not employed	1 (6.7%)
	Private sector/self-employed	4 (26.7%)
	Government sector	2 (13.3%)
	Student	2 (13.3%)
	Retired	3 (20%)
	Housewife	3 (20%)

#### Observations during pilot testing

Most patients suggested keeping ‘tapai’ instead of ‘hajur’. Both words give the same meaning i.e. ‘you’, when translated to English. So we decided to go with the participants’ preference to maintain consistency in the questionnaire.

In the sentence, “please go to next page”. Four patients suggested using ‘pristha’ instead of ‘page’. ‘Pristha’ is translated as the word ‘page’ in Nepalese language. Whereas page is translated as the word ‘page’. पेज (page) and page are pronunciation is the same. In open-ended question No 60, one patient complained about bleeding from the nose, one patient complained about dry skin and no interest in taking food.

### Quantitative results

Table 2 shows the pilot testing descriptive results and comments by patients. The mean and standard deviation was calculated for item 31 to item 59. Importantly, for all non-surgical items patients used the entire range of the response options from 1 to 4 and no missing responses were observed. The five items of the surgical sub-scale were only applicable to 5 out of 10 patients, and therefore the number of missing responses is 10. Among non-surgical items, the highest mean (indicating high symptom burden) was observed for the item number. 35 “Have you been short of breath when you climbed stairs?” (Mean = 3.33, SD = 1.11) and the lowest mean for item number. 45 “Have you been dizzy?” (Mean = 1.73, SD = 0.96).

**Table 2:** Pilot testing: Descriptive statistics and comments by patients

Item No.	Content	Response options				Missing	Mean	SD	Comments
		Not at all 1	A little 2	Quite a bit 3	Very much 4				
31	Have you coughed?	5	3	3	4	0	2.40	1.24	none
32	Have you coughed up blood?	5	2	3	5	0	1.87	1.06	none
33	Have you been short of breath when you rested?	5	2	3	5	0	2.53	1.30	none
34	Have you been short of breath when you walked?	1	5	1	8	0	3.07	1.10	none
35	Have you been short of breath when you climbed stairs?	2	1	2	10	0	3.33	1.11	none
36	Have you had a sore mouth or tongue?	6	3	3	3	0	2.20	1.21	none
37	Have you had problems swallowing?	5	4	4	2	0	2.20	1.08	none
38	Have you had tingling hands or feet?	6	3	3	3	0	2.20	1.21	none
39	Have you had hair loss?	7	4	1	3	0	2.00	1.20	none
40	Have you had pain in your chest?	6	3	3	3	0	2.20	1.21	none
41	Have you had pain in your arm or shoulder?	7	3	3	2	0	2.00	1.13	none
42	Have you had pain in other parts of your body?	5	5	2	3	0	2.20	1.15	none
43	Have you had allergic reactions?	7	3	3	2	0	2.00	1.13	none
44	Have you had burning or sore eyes?	4	3	2	6	0	2.67	1.29	none
45	Have you been dizzy?	8	4	2	1	0	1.73	0.96	none
46	Have you had splitting fingernails or toenails?	5	6	2	2	0	2.07	1.03	none
47	Have you had skin problems (e.g. itchy, dry)?	5	2	2	6	0	2.60	1.35	none
48	Have you had problems speaking?	7	2	2	4	0	2.20	1.32	none
49	Have you been afraid of tumor progression?	7	3	3	2	0	2.00	1.13	none
50	Have you had thin or lifeless hair as a result of your disease or treatment?	6	5	1	3	0	2.07	1.16	none
51	Have you worried’ about your health in the future?	5	5	3	2	0	2.13	1.06	none
52	Have you had dry cough?	5	2	1	7	0	2.67	1.40	none
53	Have you experienced a decrease in your physical capabilities?	6	3	3	3	0	2.20	1.21	Patients suggested to keep तपाई instead for हजुर to maintain consistency. Both word gives same meaning in Nepalese Language.
54	Has weight loss been a problem for you?	7	3	1	4	0	2.13	1.30	none
55	Have you had pain in the area of surgery?	3	2	–	–	10	1.40	0.55	none
56	Has the area of your wound been oversensitive?	3	1	1	–	10	1.60	0.89	none
57	Have you been restricted in your performance due to the extent of surgery?	4	1	–	–	10	1.20	0.45	none
58	Have you had any difficulty using your arm or shoulder on the side of the chest operation?	3	1	1	–	10	1.60	0.89	none
59	Has your scar pain interfered with your daily activities?	4	1	–	–	10	1.20	0.45	none

### Final version of the translated questionnaire

After the results of pilot testing and patient interviews were summarized, the problematic items and wording were changed accordingly. The report of the EORTC QLQ-LC29’s pilot testing was sent to the EORTC QoL unit for final approval and to obtain permission for culturally relevant changes in the original questionnaire—changes that would facilitate its use in Nepalese cultural settings. The translated full version of the Nepalese version of the EORTC QLQ LC-29 is available through the headquarter of the EORTC QoL Department (https://qol.eortc.org/).

## Discussion

This study presented the findings from the translation of EORTC QLQ-LC29 from English to Nepalese language and about the cultural adaptation and pilot testing of the Nepalese version of this QoL measurement tool for patients diagnosed with LC. In this paper, we report the outcome of a cross-cultural adaptation and an initial assessment of some basic distribution properties of the Nepalese version of the EORTC QLQ-LC29, which is an updated version of EORTC QLQ-LC13 developed in 1994 [11].

The module QLQ-LC 13 has been translated into more than sixty languages, and also considered as one of the standard tool used for assessing QoL in LC patients [16, 17]. The updated LC module QLQ-LC-29 is now accessible for use and can be obtained in 9 different languages through the permission of the EORTC QoL Department. The updated QLQ-LC29 module retains 12 of the 13 original LC 13 items and features new elements that illustrate the effects of targeted therapy, chemotherapy, radiotherapy, thoracic surgery, and immunotherapy [12]. The final phase 4 study has just been published recently [18].

This is the Nepalese version of LC29 which was approved and reviewed by the team of EORTC, who supervised all the steps in making this Nepalese version. This study highlights the importance of using a LC-specific instrument for measuring QOL in this patient population.

We implemented the recommended EORTC translation procedures guidelines for translating and validating the Nepalese translation of the LC29 questionnaire [15]. The final finalized translation was submitted to the EORTC where it was evaluated for the accuracy in translation as well as the method of translation. There were a small number of questions related to the translation which was discussed with the translators (FT1, FT2, BT1 and BT2) and incorporated in the final version, which has been approved by the EORTC with the requisite copyright and can now be used in various clinical trials of LC as well as in routine QoL assessment for Nepalese speaking patients. The final Nepalese translated version has been translated in a way that it is culturally appropriate as well, no culturally inappropriate terms have been used and substantial consideration was given to make the sentences generally understandable rather than a pure focus on translation, for example, the term ‘allergy’ was not translated but was written as allergy in Devanagari script as people in Nepal use allergy in daily communication than actual translation.

One limitation of this paper that the sample size was not sufficient to gain data on the performance of the new questionnaire as a tool for clinical practice or international clinical studies. To this end, future large scale trials are needed and such trials also would allow performing psychometric analysis. A similar recommendation was spelled out by Marinho et al. who recently translated the QLQ-LC29 into the Portuguese language [19, 20].

## Conclusion

In conclusion, the standardized translation procedure and the pilot testing resulted in an EORTC-approved Nepalese version of the QLQ-LC29. The accessibility of this questionnaire in Nepalese adaptation will encourage the appraisal of the health-related QoL of LC patients in Nepal, especially with regards to clinical research, yet also possibly in the clinical practice settings. Future investigations with a larger sample are needed to look at the psychometric properties of the Nepalese version of the EORTC QLQ-LC29 among patients with lung malignancy and how well the new module performs in clinical studies and clinical practice.

## Supplementary information

**Additional file 1.**

## Data Availability

The datasets used and/or analyzed during the current study are available from the corresponding author on reasonable request.

## Abbreviations

EORTC: European Organization for the Research and Treatment of Cancer
LC: Lung Cancer
QoL: Quality of Life
TC: Translation coordinator
TU: Translation Unit
FT: Forward Translator
BT: Backward Translator
