# Supplementary material for: Translation and cultural adaptation of EORTC QLQ-LC 29 into Nepalese language for lung cancer patients in Nepal
Source: J Patient Rep Outcomes. 2020 Jun 17;4:46. doi: 10.1186/s41687-020-00205-w (PMC7300167; doi:10.1186/s41687-020-00205-w)
Supplement: Supplementary file 1 — Additional file 1. [file 41687_2020_205_MOESM1_ESM.pdf]

EORTC QLQ-LC29

Patients sometimes report that they have the following symptoms or problems. Please indicate the extent to which you have experienced these symptoms or problems during the past week. Please answer by circling the number that best applies to you.

During the past week:		Not at All	A Little	Quite a Bit	Very Much
31.	Have you coughed?	1	2	3	4
32.	Have you coughed up blood?	1	2	3	4
33.	Have you been short of breath when you rested?	1	2	3	4
34.	Have you been short of breath when you walked?	1	2	3	4
35.	Have you been short of breath when you climbed stairs?	1	2	3	4
36.	Have you had a sore mouth or tongue?	1	2	3	4
37.	Have you had problems swallowing?	1	2	3	4
38.	Have you had tingling hands or feet?	1	2	3	4
39.	Have you had hair loss?	1	2	3	4
40.	Have you had pain in your chest?	1	2	3	4
41.	Have you had pain in your arm or shoulder?	1	2	3	4
42.	Have you had pain in other parts of your body?	1	2	3	4
43.	Have you had allergic reactions?	1	2	3	4
44.	Have you had burning or sore eyes?	1	2	3	4
45.	Have you been dizzy?	1	2	3	4
46.	Have you had splitting fingernails or toenails?	1	2	3	4
47.	Have you had skin problems (e.g. itchy, dry)?	1	2	3	4
48.	Have you had problems speaking?	1	2	3	4

SPECIMEN

Please go on to the next page

**During the past week:**	**Not at All**	**A Little**	**Quite a Bit**	**Very Much**
49. Have you been afraid of tumor progression?	1	2	3	4
50. Have you had thin or lifeless hair as a result of your disease or treatment?	1	2	3	4
51. Have you been worried about your health in the future?	1	2	3	4
52. Have you had dry cough?	1	2	3	4
53. Have you experienced a decrease in your physical capabilities?	1	2	3	4
54. Has weight loss been a problem for you?	1	2	3	4

**Please answer the following questions only if you had surgery for lung cancer:**	**Not at All**	**A Little**	**Quite a Bit**	**Very Much**
55. Have you had pain in the area of surgery?	1	2	3	4
56. Has the area of your wound been oversensitive?	1	2	3	4
57. Have you been restricted in your performance due to the extent of surgery?	1	2	3	4
58. Have you had any difficulty using your arm or shoulder on the side of the chest operation?	1	2	3	4
59. Has your scar pain interfered with your daily activities?	1	2	3	4

Were there any symptoms or problems that were not covered by the questionnaire, but were relevant for you in the past week?

60. ___________________________________	1	2	3	4
61.____________________________________	1	2	3	4
62.____________________________________	1	2	3	4

SPECIMEN

© Copyright 2016 EORTC Quality of life Group. All rights reserved. Phase III completed.
